# The Potential Role of fNIRS in Evaluating Levels of Consciousness

**DOI:** 10.3389/fnhum.2021.703405

**Published:** 2021-07-08

**Authors:** Androu Abdalmalak, Daniel Milej, Loretta Norton, Derek B. Debicki, Adrian M. Owen, Keith St. Lawrence

**Affiliations:** ^1^Department of Physiology and Pharmacology, Western University, London, ON, Canada; ^2^Brain and Mind Institute, Western University, London, ON, Canada; ^3^Imaging Program, Lawson Health Research Institute, London, ON, Canada; ^4^Department of Medical Biophysics, Western University, London, ON, Canada; ^5^Department of Psychology, King’s College, Western University, London, ON, Canada; ^6^Clinical Neurological Sciences, Western University, London, ON, Canada; ^7^Department of Psychology, Western University, London, ON, Canada

**Keywords:** functional near-infrared spectroscopy, motor imagery, disorders of consciousness, brain-computer interface, time-resolved fNIRS

## Abstract

Over the last few decades, neuroimaging techniques have transformed our understanding of the brain and the effect of neurological conditions on brain function. More recently, light-based modalities such as functional near-infrared spectroscopy have gained popularity as tools to study brain function at the bedside. A recent application is to assess residual awareness in patients with disorders of consciousness, as some patients retain awareness albeit lacking all behavioural response to commands. Functional near-infrared spectroscopy can play a vital role in identifying these patients by assessing command-driven brain activity. The goal of this review is to summarise the studies reported on this topic, to discuss the technical and ethical challenges of working with patients with disorders of consciousness, and to outline promising future directions in this field.

## Introduction

**Figure 1:**
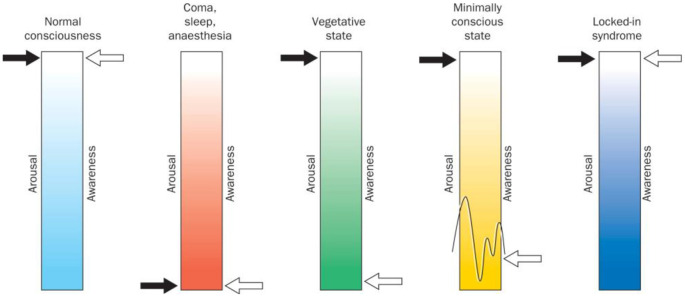
Arousal (wakefulness) vs. awareness in disorders of consciousness and locked-in syndrome. Normal consciousness is categorized by high levels of arousal and awareness. On the other hand, comatose state, sleep, and deep anaesthesia are categorised by the absence of all signs of arousal and awareness. Vegetative state (VS) is defined as wakefulness without any signs of awareness, while minimally conscious patients show high levels of arousal and experience some signs of awareness that are present albeit inconsistent. Finally, locked-in patients show high levels of arousal and awareness similar to normal consciousness but lack most physical and verbal ability to interact with their environment. Figure retrieved from Laureys et al. (2004).

Vegetative state (VS) and minimally conscious state (MCS) are clinical conditions in which patients experience impaired consciousness, with VS patients demonstrating signs of wakefulness (eye-opening and closing) but lacking all awareness of themselves and of their environment (Silva et al., 2010; Owen, 2019; see Figure 1). Indeed, repeated behavioural examination of VS patients yields no evidence of any purposeful and reproducible voluntary behavioural response to various forms of stimulation (e.g., visual, auditory or noxious; Fernández-Espejo and Owen, 2013). Hence, it is on this basis that the awareness component of consciousness is assumed to be absent. Over time, if a VS patient demonstrates inconsistent but purposeful behavioural responses, they are said to have progressed to MCS. These patients, similar to VS patients, experience sleep-wake cycles, but also retain inconsistent ability to behaviourally follow commands (Giacino et al., 2002). Differentiating between patients in a VS and an MCS is critical as MCS patients are more likely to experience pain and suffering, and may benefit from treatments aimed at improving their quality of life (Boly et al., 2008). Moreover, MCS patients are also more likely to recover higher levels of consciousness over time (Hirschberg and Giacino, 2011).

Subjective behavioural tests are the current gold-standard clinical tool used to assess consciousness at the bedside following a severe brain injury. However, detecting behavioural responses in patients with a disorder of consciousness (DOC) can be challenging, as bedside assessments rely on subjective interpretation of inconsistent motor or verbal responses, leading to a high rate of misdiagnosis (Schnakers et al., 2009; Vanhaudenhuyse et al., 2010). Schnakers et al. (2009) reported that 41% of patients classified as being VS were incorrectly diagnosed by the medical staff’s behavioural observations and were actually MCS when assessed using a standardised behavioural assessment known as the Coma Recovery Scale-Revised (CRS-R). However, even with thorough neuro-behavioural assessment scales, such as the CRS-R, objective neuroimaging assessments of residual brain function have found that around 17% of VS patients may have higher levels of consciousness than can be detected at the bedside (Monti et al., 2010). In addition, a small subgroup of patients may retain residual awareness even though they lack all physical and verbal ability to convey this (Owen et al., 2006).

Over the last 20 years, functional neuroimaging has played a key role in discerning between VS and MCS. In particular, active paradigms that require patients to regulate their brain activity in response to commands and passive paradigms, such as listening to auditory stimuli, provide objective markers that can help discriminate between these two states (Owen et al., 2006; Kondziella et al., 2016, 2020). To this end, the aim of this review is to briefly summarise the role of different imaging modalities in studies of DOC with a particular focus on the applications of functional near-infrared spectroscopy (fNIRS). fNIRS provides relatively high temporal resolution with good spatial resolution, depending on the number of channels available. The technology is safe, portable and inexpensive, making it ideal for bedside studies of DOC patients. This review will summarise and discuss the contributions of fNIRS to the assessment of brain function in DOC patients. More specifically, the focus will be on active paradigms that illicit command-driven brain activity, as most progress made has been in this area, rather than more passive and resting-state paradigms. Finally, the ethical and technical challenges, as well as promising future directions in the field, will also be discussed.

### Functional Neuroimaging as a Tool to Assess Awareness

To better appreciate the potential role of fNIRS in the study of DOC, a brief overview of the application of other neuroimaging modalities is required. Owen and colleagues first showed that functional magnetic resonance imaging (fMRI) could be used as a tool to assess residual awareness in DOC by detecting command-driven brain activity (Owen et al., 2006). They showed that a patient clinically diagnosed as being in a VS was in fact aware and able to modulate their brain activity in response to commands. More specifically, the patient was asked to imagine playing a game of tennis in the MRI scanner every time they heard the word “tennis” and to imagine navigating around their house whenever they heard the word “house”. The patient’s corresponding brain activity in the supplementary motor area (SMA) during motor imagery and the parahippocampal gyrus, posterior parietal-lobe, and lateral premotor cortex (PMC) during spatial navigation was indistinguishable from that of healthy controls performing the same tasks. These activation patterns that occurred in response to specific commands suggested that the patient was in fact aware (Owen et al., 2006). A follow-up study replicated these findings in a cohort of 23 VS patients; four of the patients were able to produce consistent and reliable brain activity in response to commands and therefore were covertly aware (Monti et al., 2010).

Subsequent to this study, various other task-based paradigms have been adopted to identify patients who are aware but misdiagnosed as being in a VS. Bekinschtein et al. (2011) used a motor imagery paradigm (i.e., imagine moving your right or left arm) to assess the preparatory movement brain activity in VS patients. Of the 24 patients, two were able to produce significant brain activity in premotor areas when asked to try to move their left or right arm, suggesting a preserved ability to follow commands. In addition to motor imagery, aspects of speech processing, such as cerebral responses to a patient’s own name and responses to other types of acoustic stimuli (e.g., words, sentences), have been investigated in VS and MCS patients (Coleman et al., 2007, 2009; Di et al., 2007; Marino et al., 2017). In the study by Di et al. (2007), 4/4 MCS and 5/7 VS patients recruited showed primary auditory cortex activation in response to hearing their own name spoken by a familiar voice. All MCS patients and 2/7 VS patients also showed activation in associative temporal areas that are responsible for higher-order linguistic processing. Moreover, both VS patients that showed the expected activation pattern progressed to MCS, suggesting that this approach could be useful in identifying patients who are likely to go on to recover from a VS. In other studies, visual paradigms ranging from passive processing of stimuli (light, colour etc.) to visual attention tasks requiring the patient to focus on particular stimuli have also been used to assess brain function and even awareness (e.g., Monti et al., 2013).

**Figure 2:**
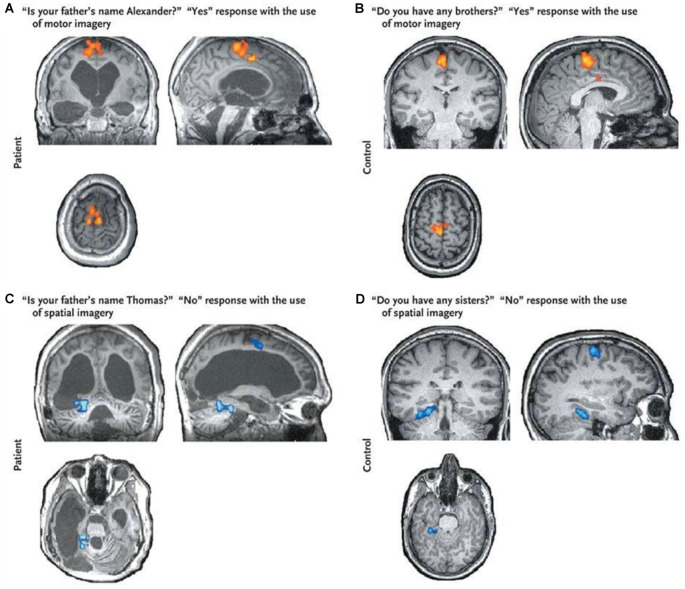
Brain responses to questions obtained from a VS patient (panels **A–C**) and that obtained from a healthy control (panel **D**). The “Yes” response shows clear activation in the supplementary motor area (SMA; shown in red) suggesting the patient was imagining playing tennis and therefore trying to answer “Yes” to the question. The “No” response shows activation in the parahippocampal gyrus (shown in blue) suggesting the patient was imagining moving around his home and therefore trying to answer “No” to the question. Brain activity from both responses was indistinguishable from that of a healthy control. This figure was retrieved fromMonti et al. (2010).

Functional MRI has also been adopted as a brain-computer interface (BCI) to establish rudimentary mental communication with DOC patients (Luauté et al., 2015). Monti et al. (2010) instructed one DOC patient to use the motor and spatial imagery to answer yes/no autobiographical questions (see Figure 2). The patient correctly answered five of six questions in the scanner, even though bedside communication remained impossible. Other work, such as that conducted by Bardin et al. (2011), used motor imagery and a multiple-choice paradigm to attempt to communicate with DOC patients. However, in that case, while one of six patients showed significant brain activity during the task, incorrect responses were obtained to both questions asked.

An alternative imaging approach for assessing residual brain function is positron emission tomography (PET) using ^18^F-fluorodeoxyglucose (FDG) to image metabolic activity in the brain (Vanhaudenhuyse et al., 2010, 2011). Previous work by Stender et al. (2014) compared the diagnostic and prognostic usefulness of FDG PET and fMRI in VS and MCS patients. Their study revealed that FDG PET had higher sensitivity in identifying MCS patients and better agreement with behavioural CRS-R scores in comparison to fMRI. However, the lower sensitivity of fMRI is not surprising, considering that mental imagery requires high-level cognitive function. In a follow-up study, Stender et al. (2016) used FDG PET to identify the minimum energetic requirement for consciousness (i.e., the presence of awareness requires 42% of normal cortical glucose metabolism). In this regard, FDG PET and fMRI provide complementary information: FDG PET indicates the minimum energy metabolism required to support neuronal activity, while fMRI provides information regarding the preservation of higher-level brain function.

Electroencephalography (EEG) has been investigated as a portable technology for assessing residual awareness at the bedside. Given its high temporal resolution (on the order of milliseconds), low cost and portability, EEG is a promising alternative to fMRI. The systems are simple and compact, allowing for bedside monitoring of brain activity. One of the earlier studies to use EEG to assess awareness was by Schnakers et al. (2008), who asked MCS patients to count the number of times they heard their name in a sequence of names. A subgroup of patients showed a larger P300 signal (which is an event-related potential elicited during decision making) when counting the occurrences of their own name, suggesting preserved cognition for autobiographical information. Later studies have attempted to replicate fMRI studies by using motor imagery to assess command-driven brain activity. A study involving a cohort of DOC patients showed that three patients clinically diagnosed as being in a VS were in fact aware as they were able to produce mental imagery responses that were decoded using EEG (Cruse et al., 2011). Further studies have attempted to compare different motor imagery and spatial navigation tasks in MCS patients and concluded that spatial navigation yielded significant results less often than motor imagery (Horki et al., 2014).

EEG has also been used in conjunction with PET to discriminate between VS and MCS patients and to predict functional outcomes after 1 year post-examination. Chennu et al. (2017) showed that the strength of EEG connectivity matched the re-emergence of behavioural awareness, with VS patients exhibiting a lack of structured connectivity over the frontoparietal networks. Furthermore, patients were categorised as either PET-positive if they had partial preservation of activity in the frontoparietal cortex and PET-negative otherwise. Comparison of the PET and EEG results showed that patients identified as PET-positive showed higher alpha and lower delta band connectivity.

Although promising, PET, fMRI, and EEG have disadvantages. PET and fMRI are expensive, which limits their accessibility to only highly specialized research centres. This hinders their widespread use, especially for repeated examinations. Moreover, the need to transport patients to imaging suites carries its own risks, which prevents frequent testing. MRI is also prone to motion artefacts, which can be challenging considering DOC patients can have difficulties lying still for extended periods (Schwarzbauer and Schafer, 2011). Additionally, patients with metallic implants cannot participate in studies, limiting the accessibility of this technique to only a subgroup of patients with brain injuries. EEG, on the other hand, allows for bedside measurements and has an excellent temporal resolution, but suffers from low spatial resolution, making it difficult to localise where the neuronal activation is originating. It can also be difficult to collect useful EEG data on patients with traumatic brain injuries who have focal skull defects, such as a craniotomy, as it can cause increases in the alpha, mu and beta rhythms, leading to what is known as the breach effect (Brigo et al., 2011).

### fNIRS in Disorders of Consciousness

In its simplest form, a fNIRS device consists of a light source and a detector placed some distance apart on the surface of the head. Using at least two wavelengths of near-infrared light, the changes in concentration of oxy- and deoxyhaemoglobin, which characterise the haemodynamic response, can be calculated. Over the last two decades, the rapid advancement in optoelectronic instrumentation, particularly with regards to commercially available multi-channel systems, has dramatically increased the application of fNIRS in a variety of neuroscience and clinical fields (Rupawala et al., 2018). State-of-the-art applications include hyperscanning methods to study brain activity from multiple participants simultaneously during social interactions, wearable systems for studies in naturalistic settings, and high-density devices for cortical mapping at spatial resolutions that rival fMRI (Eggebrecht et al., 2014; Yücel et al., 2017; Quaresima and Ferrari, 2019).

For fNIRS-based BCIs, the two most common tasks are motor imagery and mental arithmetic. The former, in particular, has been widely adopted since, unlike motor execution, motor imagery does not require intact thalamocortical tracts (Fernández-Espejo et al., 2015), making it preferable for patients with severe physical impairment. In previous fNIRS studies, classification accuracies in healthy controls have ranged from 63% to 98% (Coyle et al., 2004, 2007; Sitaram et al., 2007; Erdoĝan et al., 2019). The discrepancy between studies is likely due to multiple factors. First, a large variety of tasks have been used to elicit motor imagery activation: drawing different shapes such as circles or squares (Nagels-Coune et al., 2017), hand or arm movements (Mihara et al., 2013; Kaiser et al., 2014; Rahman et al., 2019, 2020), squeezing a ball (Coyle et al., 2004), and coordinated finger tapping (Sitaram et al., 2007; Holper and Wolf, 2011). Differences in the complexity of the task could affect classification accuracy, depending on the ability of participants to perform the task consistently. Second, there has been considerable variation in sample size, with some studies calculating overall accuracy on tests that involved the same participants in multiple sessions. Another key issue is the location of the probes on the head. For the majority of studies, optodes were placed as a grid over the entire motor cortex instead of focusing on secondary motor areas. Previous fMRI work (Boly et al., 2007) has shown that the SMA and PMC are the main areas activated during motor imagery and not the primary motor cortex. This finding indicates that overall accuracy would be increased by careful placement of the probes to maximize sensitivity to the motor planning regions. Finally, most motor imagery studies conducted to date have used standard continuous-wave (CW) fNIRS systems; consequently, classification accuracy is likely impacted by the limited depth sensitivity of NIRS (Scholkmann et al., 2014).

**Figure 3:**
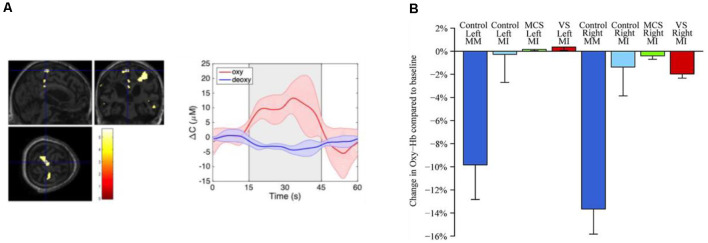
**(A)** Functional magnetic resonance imaging (fMRI) and functional near-infrared spectroscopy (fNIRS) response from a VS patient during motor imagery (retrieved from Abdalmalak et al., 2020a). fMRI (left panel) depicts increased blood oxygenation level-dependent signal in SMA which is congruent with findings from fNIRS testing (middle panel) depicting an increased concentration of oxygenated haemoglobin during motor imagery block (grey shaded area). **(B)** Change in concentration of oxyhaemoglobin during various tasks and for VS, minimally conscious state (MCS) and healthy controls (retrieved from Kempny et al., 2016).

To date, only a few fNIRS studies have used motor imagery to assess brain function in DOC patients (see Table 1). Molteni et al. (2013) compared brain activity from two MCS patients to activity acquired from a healthy control during various tasks, including somatosensory stimulation, passive movement, and active movement. In the active movement condition, patients were asked to open and close their hands, which they hypothesised would elicit brain activity associated with motor imagery due to the inability of the patients to behaviourally follow commands. Activation from channels over the motor cortex was detected for both patients during the active movement condition. However, other channels not located over motor-related regions were also activated, including channels corresponding to brain lesions as confirmed by MRI. Another study by Kempny et al. (2016) involved MCS and VS patients performing a kinaesthetic motor imagery task (i.e., imagine squeezing a ball) in a block design. Overall, no reliable activation pattern was found across the 14 patients. Five patients demonstrated the expected fNIRS response (i.e., increased oxyhaemoglobin and a concurrent decrease in deoxyhaemoglobin) during the task period, six showed inverse oxygenation (increase in deoxyhaemoglobin and a concurrent decrease in oxyhaemoglobin; see next paragraph for more details on this phenomenon), and the remaining three could not be classified into either response group. Moreover, no significant differences between VS and MCS patients were found based on these fNIRS results (see Figure 3B for a summary of the results).

**Table 1:** Summary of fNIRS studies conducted on DOC patients to date.

Source	Country	fNIRS System (wavelengths)	No. of channels	Task	Brain regions of interest	Sample size (VS/MCS)	Etiology
Molteni et al. (2013)	Italy	Commercial CW system-make not specified (760 and 830 nm)	32	Somatosensory stimulation, passive and active movements	Motor and somatosensory areas	2 (0/2)	TBI
Kempny et al. (2016)	United Kingdom	NIRScout system by NIRx Medical Technologies (760 and 850 nm)	16	Motor imagery	Primary and secondary motor areas (M1 and SMA)	16 (5/11)	ABI, TBI, brain bleeds, hydrocephalus, Aneurysm, haemorrhage
Kurz et al. (2018)	Austria	NIRScout system by NIRx Medical Technologies (760 and 850 nm)	47	Mental arithmetic	Frontal lobe	2 (0/2)	Encephalopathy, polytrauma
Abdalmalak et al. (2020a)	Canada	In-house built TR system (760 and 830 nm)	4	Motor imagery	Secondary motor areas (SMA and PMC)	6 (1/4) and 1 locked-in patient	TBI, ABI, Stroke

*VS, Vegetative state; MCS, Minimally conscious state; TBI, Traumatic brain injury; ABI, Anoxic brain injury; SMA, Supplementary motor area; PMC, Premotor cortex; M1, Primary motor area*.

Motor imagery, as a task, has its own limitations. First, the magnitude of signal change associated with motor imagery is generally lower than that caused by motor execution (Batula et al., 2017), and is dependent on the complexity of the motor imagery task (Holper and Wolf, 2011). Second, previous fMRI work has shown that motor imagery is not detectable in 10–15% of participants (Fernández-Espejo et al., 2014), which has been attributed to sensitivity issues with current imaging modalities and the inability of some participants to perform motor imagery reliably. A further challenge with motor-imagery-based fNIRS studies has been the observation of inverse oxygenation; that is, the reversal of the oxy- and deoxyhaemoglobin signals during the task period (Holper et al., 2011). This phenomenon adds to the complexity of building a generic BCI since most are designed to detect the expected response characterised by an increase in oxyhaemoglobin and a decrease in deoxyhaemoglobin. Inverse oxygenation has been attributed to a possible reduction in oxygen consumption, leading to focal deactivation (Holper et al., 2011). Alternatively, partial volume errors (i.e., activation in brain areas adjacent to the regions of interest) could be another potential cause (Abdalmalak et al., 2020b). More specifically, inadvertent movement during rest periods will lead to increased activity in the primary motor cortex adjacent to the secondary motor areas. This out-of-phase activity could be misinterpreted as decreased oxygenation during the task epochs given the relatively poor spatial resolution of fNIRS.

An alternative to motor imagery that has been developed for BCI applications is mental arithmetic, which involves covert mental calculations without the use of external aids such as a pen or paper (Naseer and Hong, 2015). Mental arithmetic activates areas of the prefrontal cortex (Artemenko et al., 2018), which are brain regions preferred in many fNIRS studies due to the practical advantage of avoiding hair. Qureshi et al. (2017) reported that the signal quality obtained with mental arithmetic is generally better than for motor imagery. Mental arithmetic has been successfully applied as a paradigm for BCI studies involving healthy participants and patients with brain injuries (Naseer and Hong, 2015). To date, the only study to use mental arithmetic to assess residual awareness involved a single MCS patient (Kurz et al., 2018). The protocol involved 10 trials of serial subtraction, each lasting 14 s, and was performed on three different days. Visual inspection of the recorded time series suggested activation in one of 10 trials in a single session; however, activation across all 10 trials did not reach statistical significance.

As mentioned earlier, these studies were conducted using CW-fNIRS systems. These systems have high temporal resolution and can incorporate a large number of source-detector pairs to improve spatial resolution. However, CW-NIRS suffers from a number of limitations. First, concentrations of oxy- and deoxyhaemoglobin cannot be quantified because the path length of photons cannot be measured. In general, this is not a major issue in functional studies since relative changes in regional oxy- and deoxyhaemoglobin concentrations are sufficient to map brain activation. A more significant challenge is the lack of depth sensitivity as CW-NIRS is inherently sensitive to the superficial tissues (scalp and skull). The low sensitivity is problematic for functional studies since haemodynamic changes in the superficial layers, whether task-evoked or spontaneous, can mask brain activity or be incorrectly classified as brain activity. Previous work has shown that sensitivity to the brain itself ranges from 1 to 9%, depending on the source-detector distance (Mansouri et al., 2010). Strangman et al. (2013) showed that even at source-detector distances as large as 6.5 cm, the sensitivity to the brain is only 22%. One approach for reducing scalp effects is to incorporate short-channel measurements (Saager and Berger, 2005; Gagnon et al., 2012). This approach requires placing additional detectors as close as possible to the emission fibre (typically a distance of 8 mm) to monitor changes in scalp haemodynamics that can be regressed from the signals recorded at larger source-detector distances. However, it is important to note that the effectiveness of short-channel regression is reduced if signal artefacts related to changes in systemic physiology are correlated with the functional task. In general, the response characteristics of scalp haemodynamics are slower than their cerebral counterparts, which helps separate the two signals. Nevertheless, developing robust classification algorithms to separate these signals effectively remains a challenging task for fNIRS (Scholkmann et al., 2014).

### Time-Resolved NIRS

An alternative approach to handling the challenges associated with scalp signal contamination is to improve the depth sensitivity of NIRS to increase the relative signal originating from the brain. One approach is to use time-resolved (TR) detection to record the arrival times of photons. TR-NIRS requires injecting pulses of light no more than a few hundred picoseconds in width, detecting a single photon arrival time, and rapidly repeating the procedure—typically in the order of 10–100 MHz—to build a distribution of times of flight (DTOF; Re et al., 2013). Since the arrival time of a photon reflects the distance it has travelled, late-arriving photons have a higher probability of having reached the brain compared to early-arriving photons that primarily interrogate superficial tissue.

A drawback with TR-NIRS is that the systems are complex compared to CW systems (Torricelli et al., 2014). This not only increases the cost of TR systems but also the overall size of the instruments, making the units less portable than their CW counterparts. Due to this complexity, TR-NIRS systems typically have a limited number of sources and detectors, and most studies have focused on the improvement to detection sensitivity for only a few activation paradigms, either motor execution (Kacprzak et al., 2007; Re et al., 2013; Milej et al., 2014; Lachert et al., 2017) or working memory (Kirilina et al., 2012; Molteni et al., 2012). The study by Kirilina et al. (2012) elegantly demonstrated the benefit of TR detection by monitoring oxygenation changes during a working memory task. They showed that extracerebral contributions to changes in the total number of photons (Δ*N*), analogous to CW-NIRS, were twice as large as for changes in the variance (Δ*V*) of the recorded DTOFs. This result agrees with the concept that higher statistical moments have greater sensitivity to late-arriving photons due to the right skewness of DTOFs (Liebert et al., 2003, 2004). This was further confirmed by Milej et al. (2020) who used a pneumatic tourniquet to impede scalp blood flow, which had substantially greater effects on Δ*N* compared to Δ*V*.

The potential advantages of TR-fNIRS for BCI applications involving motor imagery were investigated using a 4-channel portable system (Abdalmalak et al., 2017a). Given the *a priori* knowledge of the cortical regions involved with motor imagery (i.e., SMA and PMC), the probes were strategically placed over these cortical regions to detect the related activation. In a validation study involving fMRI, the sensitivity to motor imagery activation was approximately 30% greater for higher statistical moments compared to Δ*N* (Abdalmalak et al., 2017a). Moreover, motor imagery activity was detected by TR-fNIRS in 13 of 14 participants from whom brain activity was also detected by fMRI. In a follow-up study, the same approach was used as a BCI for a patient with Guillain–Barré syndrome (GBS), an acute paralytic neuropathy (Abdalmalak et al., 2017b). This patient was functionally “locked-in” as he had lost all motor control, including cranial nerve function, except for very limited eye movement. As the patient had retained all cognitive function, he was able to use motor imagery to answer clinically relevant questions. The accuracy of the fNIRS results was confirmed by repeating the same questions while the patient responded through his limited eye movement. This study was the first account of an fNIRS-based BCI used without prior patient training. More recently, this TR-fNIRS unit was used to assess residual awareness in DOC patients in long-term care facilities (see Table 1 and Figure 3A). Two patients, one clinically diagnosed as VS and the other as MCS, were able to regulate their brain activity in response to commands. These results were confirmed in independent fMRI tests involving the same motor imagery paradigm (Abdalmalak et al., 2020a).

The sensitivity and specificity of the portable 4-channel TR-fNIRS system was evaluated by having healthy participants use motor imagery to answer “yes” to a series of questions (Abdalmalak et al., 2020c). Overall, a sensitivity of 76% and a specificity of 71% were achieved, translating into an accuracy of 76%. While the sensitivity was consistent with previous studies, the low specificity was likely due to the lack of an active task for the “no” response, causing a greater number of false positives. This is an inherent limitation to using a NIRS device with a limited number of detection channels. However, this limitation will likely be overcome with rapid advances in instrumentation. Compact pulsed lasers and inexpensive detectors can reduce the footprint of TR-NIRS systems (Dalla Mora et al., 2015; Re et al., 2016; Buttafava et al., 2017) and enable full head coverage similar to conventional CW-NIRS units (Ban et al., 2021). Improved spatial coverage would provide the opportunity to test different tasks to assess awareness. For example, two active tasks, such as ones adopted from fMRI studies, could be used to improve the delineation between “yes” and “no” responses (Monti et al., 2010). High-density systems would also provide the opportunity to map functional connectivity (Smith et al., 2009).

### Technical Challenges of Using fNIRS on DOC Patients

Brain injury can be associated with many technical challenges for fNIRS data collection. First, intracranial haemorrhage in a region of interest can affect the quality of the signal or make signal acquisition impossible. Second, patients with traumatic brain injury may require a craniotomy or craniectomy, which can affect the signal quality by reducing contact between the probes and the scalp. Additionally, these patients may suffer from structural brain changes, which can affect the locations of the regions of interest. This is a major challenge considering that most fNIRS studies rely on the 10–20 international system of EEG electrode placement to guide fNIRS probe placement (Abdalmalak et al., 2020b). In these instances, it would be important to examine the patient’s anatomical CT or MRI scans to ensure the fNIRS probes are placed over the appropriate brain areas. Lastly, structural deformities can affect probe coupling to the scalp, resulting in sub-optimal data quality and significant motion artefacts. One approach to mitigate this issue is to affix the probes to the scalp using collodion (Yücel et al., 2014).

Additionally, DOC patients may have fluctuations in alertness, awareness, and attention over time; thus, one session of testing may not reflect their overall mental capacity. This adds to the complexity of assessing residual brain function and raises an important question of whether the absence of brain function could result from detection insensitivity or fluctuations in awareness, rather than reflecting the absence of residual awareness. In addition to hardware improvements, more sophisticated machine learning approaches such as artificial neural networks (ANN) could help improve the sensitivity of fNIRS (Erdoĝan et al., 2019). Considering that fNIRS is portable, it has the advantage of testing at different times of the day and on multiple days to better capture a patient’s full state of awareness.

With respect to BCI applications, most fNIRS studies of mental communication can only record the answers to simple yes/no questions without the ability to encode more complicated responses. The binary nature of current fNIRS-based BCIs is a major limitation if fNIRS is to be used in everyday life as it limits the information transfer between the patient and their caregivers. Emerging approaches such as temporally decoding responses to questions provide an opportunity to encode multiple answers by performing a mental task at a specific time window corresponding to a certain response (Nagels-Coune et al., 2017, 2020). While promising, this approach is slow as there is an inherent delay in the haemodynamic response requiring each time window to be at least a few seconds long.

Another challenge with using fNIRS as a BCI is the overall slow response, which hinders real-time communication. There is a delay in the haemodynamic response, which peaks approximately 5–6 s post-stimulus onset (West et al., 2019). Some BCI studies have focused on obtaining the highest accuracy while sacrificing speed. For example, Abdalmalak et al. used a block design of 5:30 min to communicate with a locked-in patient (Abdalmalak et al., 2017b). This block design was chosen since the goal was to confirm command-driven brain activity rather than providing real-time mental communication. For patients who do exhibit awareness, a faster BCI approach could provide rudimentary communication. Recent work has focused on detecting the initial dip in the haemodynamic response (within the first second or two) using vector phase analysis to improve speed (Hong et al., 2018; Zafar and Hong, 2018). This approach dramatically decreases the response time, enabling pseudo-real-time BCI applications.

### Clinical and Ethical Challenges of Working With DOC Patients

One of the main challenges of assessing consciousness clinically is the lack of a reliable gold standard. Behavioural assessment is the current clinical tool used at the bedside; however, the high rate of misdiagnosis has motivated the use of neuroimaging techniques as a way of complementing behavioural assessments. To date, neuroimaging testing is not part of routine clinical care and is not administered to confirm the diagnosis, primarily due to cost, complexity, and concerns regarding accuracy. False positives and false negatives can have detrimental effects when it comes to assessing awareness. The former can lead to false hope for families and prolonged unnecessary treatment in the ICU. On the other hand, false negatives can affect the quality of care and may lead to decreased interactions between the families and the patient (Peterson et al., 2015). With regards to BCI applications of mental communication, false positives can be particularly problematic. For instance, a false positive response to an end-of-life question would be catastrophic compared to a false negative response. It is therefore critical to interpreting the responses with caution. As such, no studies to date have attempted to ask end-of-life questions due to these challenges.

A promising future direction would be to combine complementary information from different modalities, particularly EEG and fNIRS, along with the results from behavioural testing to confirm the diagnosis. Since awareness can fluctuate throughout the day, it is pertinent to test patients over multiple sessions on different days to capture their true capacity for maintaining awareness. This approach, although rigorous, raises questions regarding the feasibility of frequent examinations. While fMRI is often considered the gold standard of functional neuroimaging (Cui et al., 2011), it is not well-suited for repeat sessions, making bedside techniques, such as fNIRS and EEG, the primary choice for long–term testing. As a result, fNIRS and EEG could be used as screening tools to identify patients with residual brain function who could undergo subsequent testing with fMRI or PET for confirmation.

### Conclusion

The goal of this review was to summarize the current state of fNIRS in assessing awareness in DOC patients. While the application of fNIRS in this domain has been limited, the benefits of supplementing behavioural assessments with bedside monitoring of brain activity are undoubtedly clear. With continued advancements in hardware and data processing pipelines, some of the pressing technical challenges will likely be overcome, providing a truly safe, portable and inexpensive BCI for DOC patients.

## Author Contributions

AA and KStL contributed to the conception of this review. AA conducted the literature search and wrote the first draft of the manuscript. DM and KStL provided feedback and suggestions on sections “fNIRS in Disorders of Consciousness” and “Time-Resolved NIRS”. DD, LN, AO, and KStL provided critical feedback on sections “Functional Neuroimaging as a Tool to Assess Awareness”, “Technical Challenges of Using fNIRS on DOC Patients”, and “Clinical and Ethical Challenges of Working With DOC Patients”. AO and KStL secured funding for this work. All authors contributed to the article and approved the submitted version.

## Conflict of Interest

The authors declare that the research was conducted in the absence of any commercial or financial relationships that could be construed as a potential conflict of interest.
